# Assessment of Ismailia Canal for irrigation purposes by water quality indices

**DOI:** 10.1007/s10661-022-10350-y

**Published:** 2022-10-10

**Authors:** Amany S. Amer, Walaa S. Mohamed

**Affiliations:** Biology and Environmental Indicators Department, Central Laboratory for Environmental Quality Monitoring (CLEQM), National Water Research Center (NWRC), Cairo, Egypt

**Keywords:** Ismailia Canal, Irrigation water, Irrigation criteria, Water quality index

## Abstract

Ismailia Canal is one of the significant streams of the Nile River in Egypt. The study aimed to determine the water quality of Ismailia Canal based on the regional and seasonal variability of physicochemical parameters, irrigation criteria, and the irrigation water quality index (IWQI). It was observed that the physicochemical parameters were within the acceptable FAO irrigation limits. All cations and anions values were within the acceptable FAO limits for irrigation, except the potassium (K^+^) concentrations were over the permissible irrigation limits. The one-way analysis of variance (ANOVA) suggested a significant seasonal variation in the canal’s water quality concerning all parameters (*p* value ˂ 0.05). However, the regional variation among various sites was statistically insignificant (*p* value > 0.05). Statistical analysis was used to calculate the correlation coefficient between different parameters, and the study showed highly significant correlation coefficients between different pairs of water quality parameters. The correlation matrix showed that the pH significantly affected IWQI (*r* = 0.661). The irrigation criterion values for Ismailia Canal were good, and the WQI levels for irrigation utilization at all studied sites were satisfactory. Deterioration of water quality may occur due to industrial, municipal, and agricultural activities. Drainage water should be treated before being mixed with irrigation water to improve its suitability for irrigation.

## Introduction

The Nile River is Egypt’s main supply of fresh water. Recently, Egypt has been experiencing water scarcity caused by various factors. The most well-known factors are ineffective irrigation systems and water waste. Egypt’s water consumption is rising due to rising population, rapid economic growth, and environmental destruction. The quality and scope of human-related activities in a region's basins substantially impact surface water quality. Wastewater discharges that have not been properly regulated have an immediate and long-term impact on consumers’ health (Goher et al., 2014). Because of the high concentration of organic pollutants that are hazardous to the aquatic environment and affect the health of the flora and fauna, the discharge of effluent without sufficient treatment greatly impacts the ecosystem (Oyekanmi et al., 2021).

In 1862, the construction of Ismailia Canal was completed providing Egypt with vital irrigation and drinking water (Goher et al., 2014). The canal begins at the Nile inlet in Cairo and continues east to the Ismailia governorate, passing through Cairo’s governorates. Port Said (90 km) and Suez (about 80 km) governorates are served by separate arms of the river, which separates near Ismailia town. The total area served by the river is approximately 108,200 fedden (Geriesh et al., 2008). It is used for irrigation, domestic, and industrial purposes, as well as a primary source of drinking water for many Egyptians, including those in the northern part of Great Cairo, Shubra El-Kheima, El-Amira, Mattaria, Musturod, Abu-Zaabal, Inchas, Belbeis, Abu-Hammad, Zagazig, and El-Tal el Kabeer.

Ismailia Canal is the furthest downstream from the main Nile River. Its water quality is susceptible to numerous sources of contamination. Shubra El-Kheima, Musturod, and Abu Zaabal are the three main industrial zones in Egypt, located upstream of Cairo on the western side of Ismailia Canal. Alum (aluminum sulfate), Abu Zaabal Fertilizers company, and a detergent business are located in these locations. Wastewater treatment plants discharge high levels of aluminum, iron, and manganese wastewater into the surrounding environment, causing significant changes in pH and chemical properties. There is much pollution from nearby communities and septic tanks and agricultural effluents; seep into the canal. Contaminated water poses a serious health risk due to reusing drainage water and pumping filthy groundwater. Many organic pollutants are known to be harmful or carcinogenic. Surface and ground waters must be studied for changing component levels to reduce pollution and enhance water quality (El-Amier et al., 2021).

It is important to consider the irrigation water’s salinity and ion toxicity before using it. When there is too much salt in the soil (sodicity), the soil structure breaks down. Waterways must be regularly monitored to detect any shifts in salt concentration. Plant growth can be affected by high levels of HCO_3_^−^, Cl^−^, Na^+^, Mg^++^, and other trace elements (Shrestha & Kazama, 2007). Heavy metals are regarded as dangerous and toxic when present in water bodies. Heavy metal contamination in wastewater can cause diseases like cancer, skin mutations, and mental disorders when it is deposited and consumed. To promote a healthy ecosystem, it is crucial to remove these metals from wastewater before releasing them into the environment (Adeleke et al., 2017).

The water quality index (WQI) is one of the best standard methods to assess a certain area’s overall water quality based on several different criteria (Kothari et al., 2021). To achieve the final score of WQI, many factors related to water quality are taken into account. It is one of the most effective ways of disseminating information regarding water quality (Cabassud et al., 2001). With the help of WQI, the general public, politicians, and other judges can quickly and easily learn about water quality in their area of interest (Rothmaier et al., 1997). Water resources in a community can be managed and exploited in various ways depending on the quality of the water (Kothari et al., 2021). Consequently, this research aims to investigate Ismailia Canal water quality for irrigation based on the regional and seasonal variability of physicochemical parameters, irrigation criteria, and the irrigation water quality index (IWQI).

## Materials and methods

### Description of the study area

**Fig. 1:**
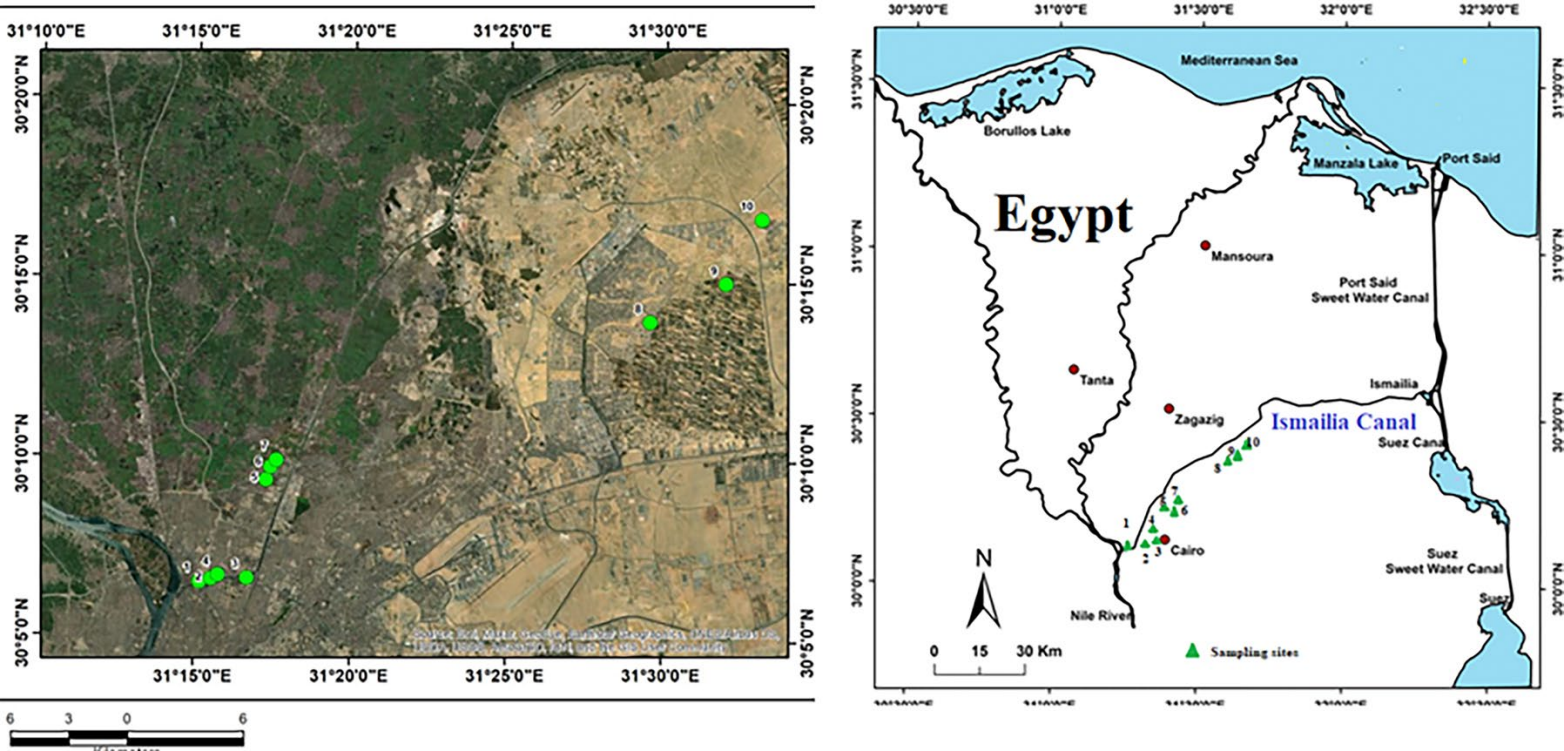
Ismailia Canal sampling locations are shown on the map. (On the right side, the general position of the canal in Egypt is depicted)

Ismailia Canal is one of the most important irrigation canals in Egypt. It was constructed to transport fresh water from River Nile in north Cairo (El-Mezalat) to Ismailia, Port Said, and Suez governorates (Fig. 1). It is 128 km long, 2.1 m in depth, and 18 m in width. The water canal’s flow rate is 433.56 m^3^/s. About 108.200 fedden are covered by the canal, which provides water for drinking and agriculture and is used in industrial processes (Goher et al., 2014).

### Collection of water samples

The research samples were collected in polyethylene bottles from ten sites along Ismailia Canal and transferred to the laboratory in an ice box within 6 h of collection. Each location had three replicates taken in the same line: right, center, and left canal side. The water samples were collected in 2020 during the research period (winter, spring, summer, and autumn). Table 1 and Fig. 1 depict the locations of the sites.

**Table 1:** Location of studied sites of Ismailia Canal

Site No.	Sites	Latitude	Longitude
1	El-Mazalat- Mouth of Ismailia Canal	30° 06ʹ 30″	31° 15ʹ 10″
2	Before Cairo Electricity Company	30° 06′ 35″	31° 15′ 33″
3	Discharging point of Cairo Electricity Company	30° 06′ 37″	31° 16′ 42″
4	After Cairo Electricity Company	30° 06′ 41″	31° 15′ 46″
5	Before Misr Petroleum Company	30° 09′ 22″	31° 17′ 17″
6	Discharging point of Misr Petroleum Company	30° 09′ 43″	31° 17′ 25″
7	After Misr Petroleum Company	30° 09′ 55″	31° 17′ 36″
8	Before Abu Zaabal fertilizer Company	30° 13′ 52″	31° 29′ 33″
9	Discharging point of Abu Zaabal fertilizer Company	30° 14′ 58″	31° 31′ 58″
10	After Abu Zaabal fertilizer Company	30° 16′ 46″	31° 33′ 06″

### Water quality characterization

#### Field measurements

At the sampling site, pH, electrical conductivity (EC), and dissolved oxygen (DO) were measured using pH meter (ORION) model 420A and electrical conductivity meter (WTW) (Inolab) level 1, respectively.

#### Laboratory analysis

The physicochemical parameters (total dissolved solids (TDS), cations (calcium (Ca^++^), magnesium (Mg^++^), sodium (Na^+^), and potassium (K^+^)), anions (chloride (Cl^−^), nitrate (NO_3_^−^), sulfate (SO_4_^−−^), bicarbonate (HCO_3_^−^), and carbonate (CO_3_)), heavy metals (Al^+++^, Cu^++^, Pb^++^, Zn^++^, Cr^++^, and Fe^++^), and biological parameters (biological oxygen demand (BOD), fecal coliform (FC), and *E. coli*) were analyzed in the laboratory using American Public Health Association (APHA) standard procedures (Grant & Greene, 2012). Dissolved oxygen was measured using dissolved oxygen meter (WTW) model OXI 315i and oxygen sensor (WTW) model DuroX325. The COD was measured using COD spectrophotometer (TURNER) model 690 with a COD reactor (HACH). The BOD was measured using BOD manometer (WTW) (Oxitop system) 12-bottle set. The anions were measured by ion chromatography (IC), model DX-500 chromatography system. In addition, the cations and heavy metals were measured by ICP-OES instrument (Inductively Coupled Argon Plasma-Optical Emission Spectroscopy) (Perkin Elmer Optima 3000 Redial, USA). All bacterial parameters were examined by using the membrane filter technique.

#### Irrigation water quality criteria

The presence of undesired elements determines the water’s appropriateness for irrigation. To assess the quality and irrigation suitability of these waters, the most frequently calculated irrigation criteria have been used. The following formulas were used to calculate the sodium adsorption ratio (SAR), residual sodium carbonate (RSC), sodium percentage (Na%), permeability index (PI%), magnesium hazard percentage (MH%), and Kelly’s index (KI), and their categories are described in Table 2.

**Table 2:** Classification of water quality criteria and IWQI (El-Amier et al., 2021)

Criteria	Range	Class	References
Sodium adsorption ratio (SAR)	< 10	Excellent	(Oster & Sposito, 1980)
	10–18	Good	
	19–26	Fair Poor	
	> 26	Unsuitable	
Residual sodium carbonate (RSC) mmole L^−1^	< 1.25	Good	
	1.25–2.50	Medium	(Murtaza et al., 2021)
	> 2.50	Unsuitable	
Sodium percentage (Na%)	< 20%	Excellent	(Ravikumar et al., 2011)
	20%–40%	Good	
	40%–60%	Permissible/Safe	
	60%–80%	Doubtful	
	80%	Unsuitable	
Permeability index (PI%)	> 75%	Suitable	(Das & Nag, 2015)
	25–75%	Moderate	
	< 25%	Unsuitable	
Magnesium hazard percentage (MH%)	< 50%	Suitable	(Zhang et al., 2021)
	> 50%	Unsuitable	
	> 1	Unsuitable	
Kelly’s index (KI)	< 1	Suitable	(Shah et al., 2019)
	> 1	Unsuitable	
Irrigation water quality index (IWQI)	0–25	Excellent	(Şener et al., 2017)
	26–50	Good	
	51–75	Poor	
	76–100	Very poor	
	> 100	Unsuitable	

Sodium adsorption ratio (SAR) = *Na*^+^ */ √((Ca*^++^ + *Mg*^++^*) / 2)* (Richards, 1954)

Residual sodium carbonate (RSC) mmole L^−1^ = *[CO*_*3*_^*−−*^ + *HCO*_*3*_^*−*^*]—[Ca*^++^  + *Mg*^++^*]* (Murtaza et al., 2021)

Sodium percentage (Na%) = *(Na*^+^ + *K*^+^*) / (Na*^+^ + *Ca*^++^ + *Mg*^++^ + *K*^+^*)* × *100* (Oster & Sposito, 1980)

Permeability index (PI%) = *(Na*^+^ + *√HCO*_*3*_^*−*^*) / (Ca*^++^ + *Mg*^++^ + *Na*^+^*)* × *100* (Eyankware et al., 2018)

Magnesium hazard percentage (MH%) = *Mg*^++^ */ (Ca*^++^ + *Mg*^++^*)* × *100* (Zhang et al., 2021)

Kelly’s index (KI) = *Na*^+^ */ (Ca*^++^ + *Mg*^++^*)* (Shil et al., 2019)

#### Irrigation water quality index (IWQI)

The weighted arithmetic index method (Brown et al., 1972) has been used to calculate the WQI. Many scientists have relied on this mathematical method (Balan et al., 2012; Tyagi et al., 2013). The steps to arrive at a single WQI score included identifying the parameters, classifying them, assigning a relative weight to each, and then merging all the results (Şener et al., 2017; Taloor et al., 2020). Table 11 shows the factors utilized in irrigation water quality that were the most important in the selection process. The following equation was used to determine the parameter (S_i_) scores:

$${S}_{i}={V}_{actual}-{V}_{idea}/{V}_{standard}-{V}_{idea}\times 100$$

where *V*_*actual*_ is the monitored value of the n parameter and *V*_*ideal*_ is the ideal value for the parameter in pure water. Dissolved oxygen is 14.6 mgL^−1^, pH is 7, and all other values are identical to 0 (Boah et al., 2015). The Food and Agriculture Organization (FAO) (Lupien, 1994) has established a standard acceptable value for *Standard*. Furthermore, the following equation was used to determine the relative weight (*Rw*_*i*_).

$${Rw}_{i}={W}_{i}/\sum {W}_{i}$$

where *W*_*i*_ was the weight of each parameter. Each parameter was assigned a weight according to the following equation:

$${W}_{i}=1/{V}_{standard}$$

Finally, the mathematical formula of the IWQI was given by the following equation:

$$\mathrm{Irrigation\ water\ quality\ index }\left(\mathrm{IWQI}\right)=\sum {\mathrm{S}}_{\mathrm{i}}\times {Rw}_{i}$$

### Statistical analysis

The mean values, standard deviations, and the range of results were performed using Excel-Stat software. The Statistics Package for Social Science (SPSS software Version 20) was used to examine water quality data. In addition, the water quality data were tested for normality with the Shapiro–Wilk test. The relationship between the water quality indices was determined using the two-tail Spearman rank correlation. A correlation coefficient (*r*) spans from − 1 to + 1, a numerical value. Generally, the correlation between the parameters is strong when it is between 0.8 and 1 and moderate when it is between 0.5 and 0.8. One-way ANOVA was used to investigate the significant spatial and seasonal difference at a probability level of 0.05 (Faruque et al., 2005). Box plots were performed using SPSS to show a significant seasonal difference as per ANOVA.

## Results and discussion

### Water quality characterization

**Fig. 2:**
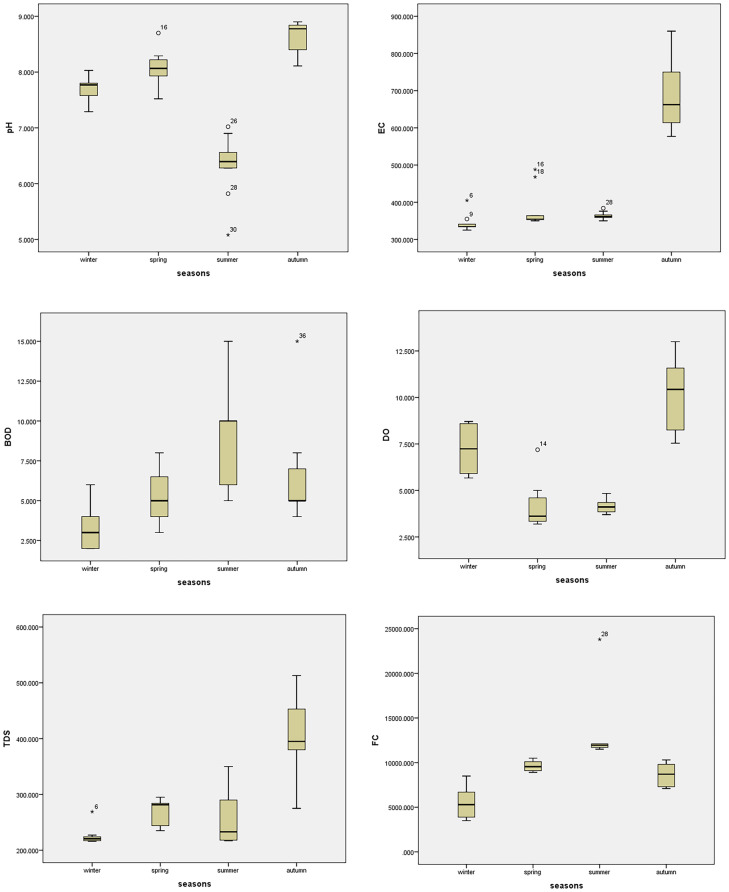

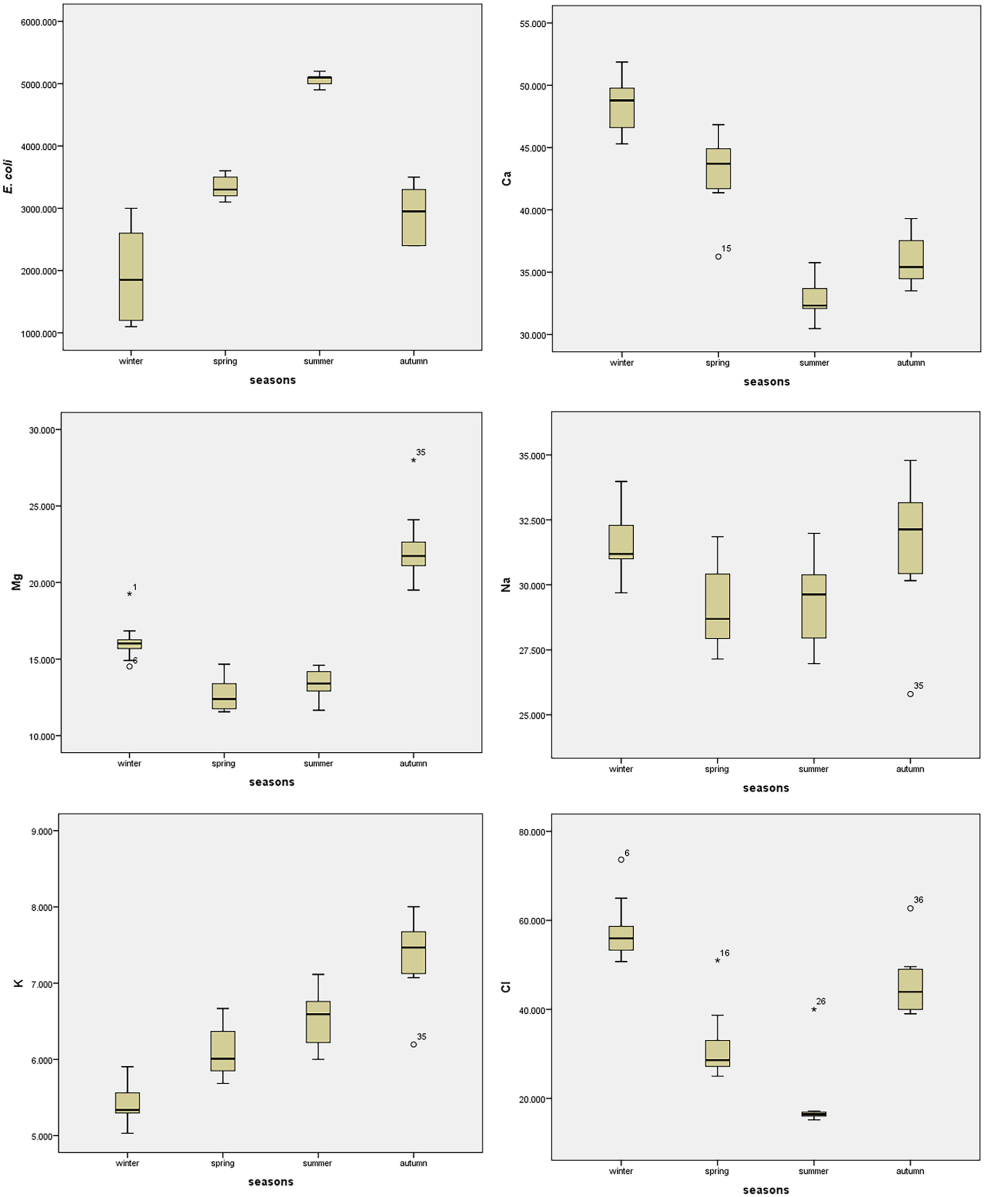

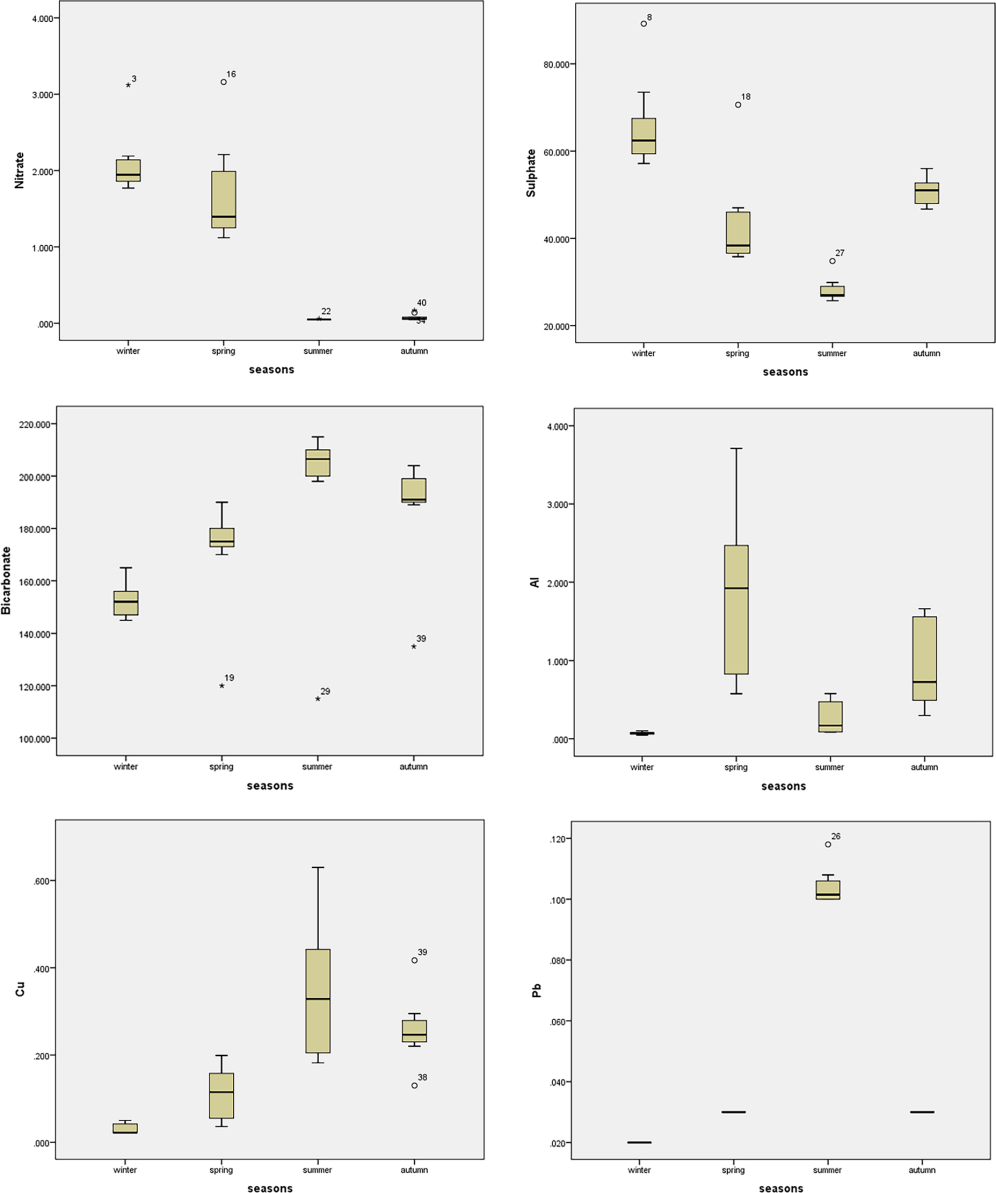

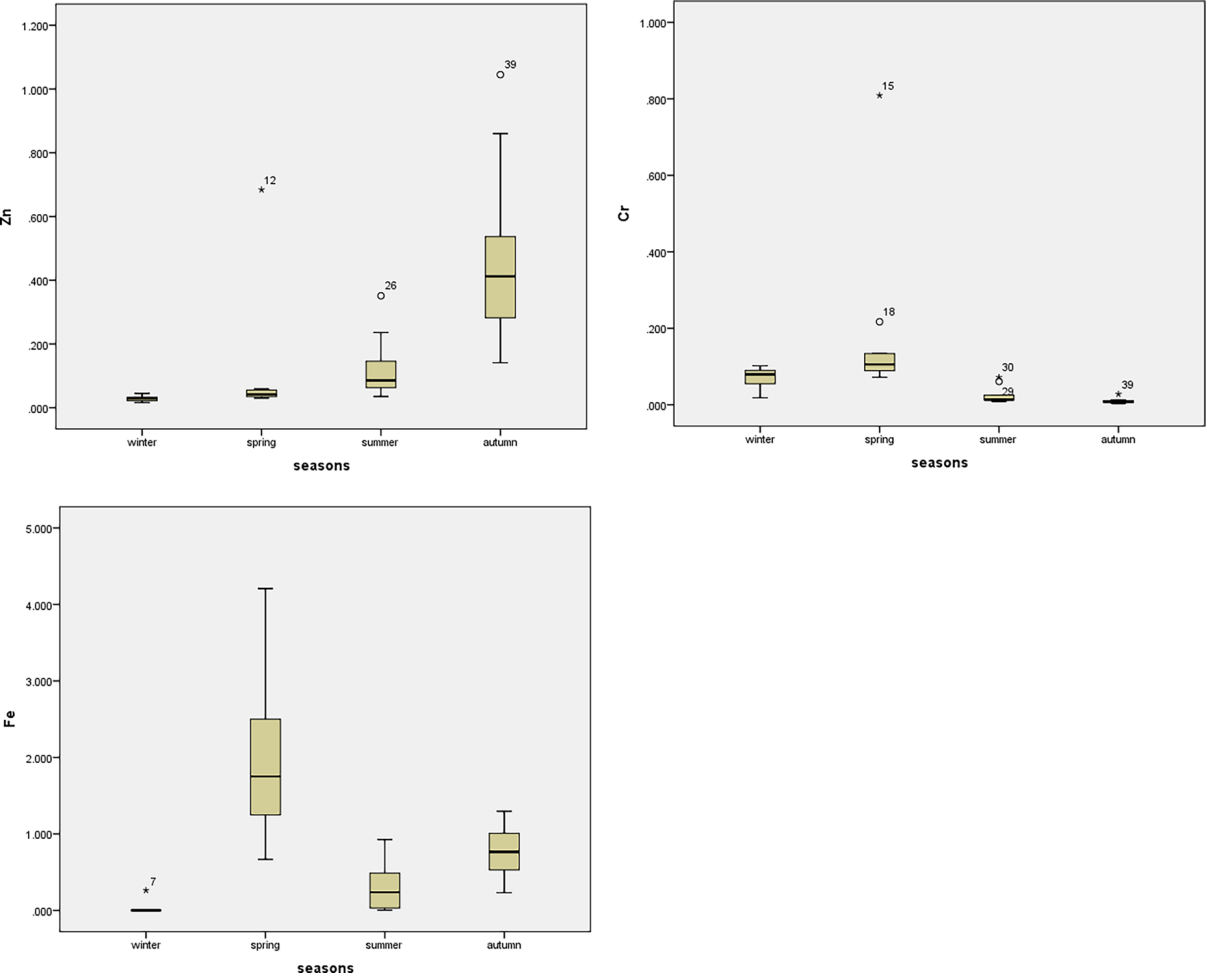
Boxplots of the parameters show statistically significant seasonal variation

The water quality of Ismailia Canal determined many essential physicochemical parameters at ten different sites during various seasons, as shown in Tables 3, 4, and 5. In addition, ANOVA values of spatial and seasonal variation of water quality parameters were given in Tables 6 and 7, respectively. Box plots for the parameters showed seasonal and spatial statistically significant differences as per ANOVA, as represented in Fig. 2. The pH is an important metric that indicates the acceptability of water for various applications, and it is one of the physicochemical parameters chosen for water quality. The pH levels in this study ranged from 7.18 to 8.09 demonstrated the water’s alkaline nature. The slight increase in the pH at some sites may be due to the inputs from industrial wastewater. The pH alterations are connected to the changes in conductivity and bicarbonate content (Roy & Rhim, 2021). It has been found that pH did not vary significantly among sites (*p* value = 0.949) (Table 6). However, pH varied significantly between seasons (*p* value = 0.00) (Table 7). It is possible to use the electrical conductivity (EC) value to indicate the total amount of dissolved salts in water. The studied sites met the FAO’s irrigation water EC standards of 3,000 μS cm^−1^ based on their EC readings. Ben-Gal et al. (2008) obtained similar observations in their studies on different water bodies (Ben-Gal et al., 2008). The variation of EC between ten sites was not statistically significant (*p* value = 0.999); however, there was statistically significant variation among various seasons (*p* value = 0.00). As shown in Table 8, the EC had a significant negative correlation with *E. coli* and a significant positive correlation with DO, TDS, BOD, Ca^++^, Na^+^, and K^+^.

**Table 3:** Descriptive statistics of physicochemical parameters of the studied sites

The sites		Parameters						
		pH	EC μS cm^−1^	DO mg L^−1^	TDS mg L^−1^	BOD mg L^−1^	FC *100 cfu 100 mL^−1^	E. coli *100 cfu 100 mL^−1^
Site 1	Mean	8.00	454.50	5.80	243.25	5.50	93.38	34.37
	Std. deviation	0.81	220.11	2.29	24.72	0.71	22.09	12.76
Site 2	Mean	7.86	423.75	6.54	279.50	4.50	100.13	36.51
	Std. deviation	0.95	151.16	3.76	86.98	2.65	13.55	9.90
Site 3	Mean	7.79	408.50	5.27	273.25	4.50	90.25	37.24
	Std. deviation	1.02	113.10	2.00	56.96	1.73	30.88	9.90
Site 4	Mean	7.72	422.00	6.54	277.50	3.75	95.38	35.03
	Std. deviation	1.04	145.85	2.49	79.79	1.71	24.25	12.76
Site 5	Mean	7.77	415.25	5.52	275.00	5.50	96.75	35.43
	Std. deviation	1.05	132.95	1.55	79.30	3.32	16.70	11.33
Site 6	Mean	8.09	483.50	6.87	320.25	9.75	79.63	29.68
	Std. deviation	0.86	149.51	3.71	79.00	6.08	31.40	15.29
Site 7	Mean	7.52	476.25	7.08	309.00	5.75	81.25	30.37
	Std. deviation	0.81	256.23	3.58	139.37	3.50	33.75	16.39
Site 8	Mean	7.23	485.75	7.09	317.50	7.75	115.88	31.91
	Std. deviation	1.03	183.89	3.98	96.59	1.71	82.78	14.15
Site 9	Mean	7.75	419.00	6.44	275.50	7.50	86.00	31.88
	Std. deviation	0.99	120.78	2.82	75.55	2.89	35.18	15.71
Site 10	Mean	7.18	436.75	6.97	305.25	5.75	82.13	30.69
	Std. deviation	1.45	159.59	4.04	118.50	2.87	35.47	16.75

**Table 4:** Descriptive statistics of cations and anions of the studied sites

The sites		Cations mg L^−1^				Anions mg L^−1^			
		Ca^++^	Mg^++^	Na^+^	K^+^	Cl^−^	NO_3_^−^	SO_4_^−−^	HCO_3_^−^
Site 1	Mean	40.05	15.86	30.41	6.35	34.02	0.79	42.37	183.25
	Std. deviation	8.29	4.09	1.72	1.00	17.17	0.91	13.99	23.71
Site 2	Mean	40.44	16.10	30.70	6.48	32.93	0.80	42.89	182.50
	Std. deviation	9.75	3.48	2.33	1.10	15.44	0.91	14.24	23.84
Site 3	Mean	38.98	16.56	29.52	6.10	35.22	1.37	44.37	182.00
	Std. deviation	7.81	5.22	1.66	0.92	18.23	1.55	16.09	26.19
Site 4	Mean	40.36	15.44	30.64	6.40	35.89	0.85	43.52	185.50
	Std. deviation	8.75	4.11	2.33	1.03	17.66	0.92	14.46	26.13
Site 5	Mean	37.43	18.31	28.25	5.90	37.43	0.83	44.62	188.25
	Std. deviation	5.36	6.48	2.83	0.27	17.74	0.91	14.27	18.41
Site 6	Mean	40.42	15.65	30.69	6.41	56.84	1.37	48.80	182.75
	Std. deviation	6.47	4.34	1.00	0.68	14.54	1.56	18.39	25.50
Site 7	Mean	40.44	15.08	30.78	6.42	36.08	0.98	45.71	179.00
	Std. deviation	7.44	4.48	2.95	1.14	16.52	1.08	12.54	25.78
Site 8	Mean	41.88	15.98	31.90	6.79	39.45	0.83	61.43	181.00
	Std. deviation	7.85	4.82	1.76	0.87	16.21	0.92	25.03	25.88
Site 9	Mean	40.09	16.58	30.47	6.23	39.26	0.93	45.19	130.25
	Std. deviation	5.40	3.91	2.67	0.87	20.39	1.05	16.05	16.24
Site 10	Mean	40.90	15.77	31.10	6.49	37.18	0.87	48.55	182.75
	Std. deviation	5.41	4.08	2.14	0.57	17.27	0.92	16.76	23.31

**Table 5:** Descriptive statistics of heavy metals of the studied sites

The sites		Heavy metals mg L^−1^					
		Al^+++^	Cu^++^	Pb^++^	Zn^++^	Cr^++^	Fe^++^
Site 1	Mean	0.26	0.14	0.05	0.13	0.03	0.41
	Std. deviation	0.24	0.09	0.04	0.17	0.03	0.43
Site 2	Mean	0.31	0.17	0.05	0.28	0.05	0.67
	Std. deviation	0.34	0.17	0.04	0.30	0.04	0.59
Site 3	Mean	0.49	0.22	0.05	0.12	0.05	0.78
	Std. deviation	0.57	0.22	0.04	0.10	0.06	1.11
Site 4	Mean	0.39	0.19	0.05	0.15	0.05	0.51
	Std. deviation	0.38	0.12	0.04	0.19	0.05	0.63
Site 5	Mean	0.77	0.12	0.05	0.14	0.23	0.23
	Std. deviation	0.99	0.11	0.04	0.21	0.39	0.31
Site 6	Mean	1.28	0.27	0.05	0.32	0.04	0.86
	Std. deviation	1.65	0.26	0.05	0.39	0.03	1.23
Site 7	Mean	1.09	0.18	0.05	0.11	0.05	1.20
	Std. deviation	0.95	0.15	0.04	0.11	0.04	0.97
Site 8	Mean	0.99	0.10	0.05	0.07	0.08	0.59
	Std. deviation	1.11	0.08	0.04	0.05	0.10	0.55
Site 9	Mean	1.27	0.28	0.05	0.34	0.07	1.43
	Std. deviation	1.41	0.19	0.04	0.48	0.03	1.90
Site 10	Mean	0.96	0.20	0.05	0.18	0.07	0.92
	Std. deviation	0.81	0.12	0.04	0.24	0.04	0.93

**Table 6:** ANOVA values represent the spatial variation of water quality parameters

Parameters	*df*	*F*	Sig
pH	9	0.35	0.949
EC	9	0.126	0.999
DO	9	0.177	0.995
TDS	9	0.303	0.968
BOD	9	1.411	0.227
FC	9	0.344	0.952
*E. coli*	9	0.151	0.997
Ca^++^	9	0.102	0.999
Mg^++^	9	0.152	0.997
Na^+^	9	0.77	0.645
K^+^	9	0.294	0.971
Cl^−^	9	0.625	0.767
NO_3_^−^	9	0.161	0.997
SO_4_^−−^	9	0.458	0.891
HCO_3_^−^	9	2.02	0.072
Al^+++^	9	0.679	0.722
Cu^++^	9	0.511	0.855
Pb^++^	9	0.005	1
Zn^++^	9	0.524	0.846
Cr^++^	9	0.773	0.642
Fe^++^	9	0.555	0.822

**Table 7:** ANOVA values represent the seasonal variation of water quality parameters

Parameters	*df*	*F*	Sig
pH	3	72.68	0.00
EC	3	92.02	0.00
DO	3	45.92	0.00
TDS	3	26.63	0.00
BOD	3	9.82	0.00
FC	3	19.45	0.00
*E. coli*	3	98.20	0.00
Ca^++^	3	100.04	0.00
Mg^++^	3	86.69	0.00
Na^+^	3	5.89	0.00
K^+^	3	46.55	0.00
Cl^−^	3	53.32	0.00
NO_3_^−^	3	77.46	0.00
SO_4_^−−^	3	44.25	0.00
HCO_3_^−^	3	9.60	0.00
Al^+++^	3	18.96	0.00
Cu^++^	3	26.79	0.00
Pb^++^	3	1819.98	0.00
Zn^++^	3	12.25	0.00
Cr^++^	3	4.69	0.01
Fe^++^	3	20.56	0.00

**Table 8:**
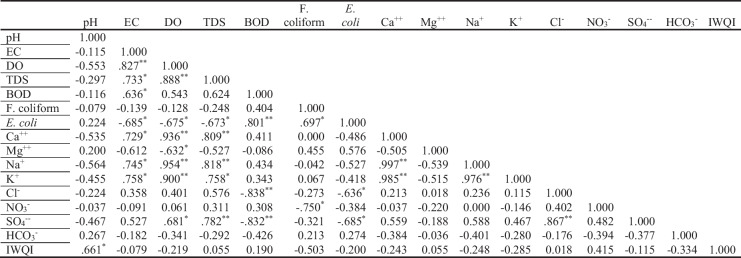
Matrix of correlation coefficients for the examined area’s water quality measures and IWQI

*Correlation is significant at the 0.05 level (2-tailed); **Correlation is significant at the 0.01 level (2-tailed)

The distribution of flora and fauna is regulated by dissolved oxygen concentration (DO). According to the current study, dissolved oxygen concentration varied between 5.27 and 7.09 mg L^−1^. Seasonal variation had significant difference (*p* value = 0.00). This finding was in agreement with Ben-Gal et al. (2008). The DO values decreased in summer due to high temperature and the high decomposition rate of organic matters (Fig. 2). The DO had a significant negative correlation with *E. coli* and Mg^++^ and a positive correlation with TDS, Ca^++^, Na^+^, K^+^, and SO_4_^−−^.

It is widely accepted that total dissolved solids (TDS) are an essential metric for assessing water quality since it is directly associated with and affected by increased turbidity, hardness, alkalinity, and conductivity. The TDS showed differences ranging from 243.25 to 320.25 mg L^−1^. The TDS was found to vary significantly between studied seasons (*p* value = 0.00). Moreover, the TDS had a significant negative correlation with *E. coli* and a positive correlation with Ca^++^, Na^+^, K^+^, and SO_4_^−−^.

The biochemical oxygen demand (BOD) is a metric for determining the amount of organic matter in water bodies. Many studies investigated that contaminated water has increased BOD levels. In the present study, spatial variation in BOD did not exist (*p* value = 0.227). Seasonal variation showed statistical significant (*p* value = 0.00). The BOD concentration ranged from 3.75 to 9.75 mg L^−1^ indicating eutrophication of the water body. The BOD had a negative correlation with Cl^−^ and SO_4_^−−^, besides a significant positive correlation with *E. coli*.

Potential human health problems are indicated by the presence of bacteria in surface water (Haque et al., 2019). The bacterial pollution of Ismailia Canal’s surface water was investigated during the study period by detecting the fecal coliform and *E. coli* levels (Table 3). The elevated coliform bacteria counts were attributed to the research area’s rapid population growth, which was facilitated by the discharge of domestic trash containing feces into city sewers. Furthermore, temperature and seasonal variations are important determinants of bacterial growth (White et al., 1991). As the temperature rises in the summer, the fecal coliform concentration also rises, due to many factors, such as the lack of water and the high organic matter concentration. Restricted water, high organic matter, and growth-supporting nutrient concentrations are necessary for a healthy ecosystem (Haque et al., 2019). The cold winter climatic conditions did not promote bacterial growth, resulting in a lower coliform count throughout the season (Tiefenthaler et al., 2008). The elevated counts could be attributed to high nutrient concentrations in the companies’ discharges. The ANOVA results were reported that there was a statistically significant difference between seasons concerning FC (*p* value = 0.00) and *E. coli* (*p* value = 0.00) (Table 7). The fecal coliform had a significant inverse relationship with NO_3_^−^ and a positive relationship with *E. coli*. Additionally, *E. coli* had a significant negative correlation with Cl^−^ and SO_4_^−−^.

Major ion concentrations control the basic hydrochemical properties of surface water (Zhang et al., 2019). All the Ca^++^, Mg^++^, and Na^+^ ions values were within the acceptable FAO limits for irrigation (Table 4). Due to salt concentration, a high Na^+^ content in irrigation water can be detrimental to crop production. All potassium (K^+^) concentrations measured at the study sites were over the FAO permitted level (2 mg L^−1^), posing a risk to vegetable production. Furthermore, the Ca^++^ ion concentration positively correlated with Na and K^+^ ions concentrations. The Na^+^ positively correlated with K^+^.

On the other hand, high Cl^−^ ions impact plant growth by increasing osmotic pressure, inhibiting crop growth, and decreasing plant water availability. The excess absorbed Cl^−^ ions in plant tissues accumulate in leaves, causing leaf burns, whereas using an excessive amount of NO_3_^−^ decreases the crop yield and quality. The ion concentrations (Cl^−^, NO_3_^−^, SO_4_^−−^, HCO_3_^−^, and CO_3_^−^) analysis indicated the allowed limits for irrigation. The Cl^−^ concentration was positively correlated with SO_4_^−−^. The highest averages of Cl^−^ and NO_3_^−^ concentrations (56.84 and 1.37 mg L^−1^, respectively) were recorded at site 6 (Discharging point of Misr Petroleum Company). High values of Cl^−^ and NO_3_^−^ at this site may be due to the mix of industrial effluent with Ismailia Canal water. ANOVA results suggested a statistically significant seasonal variation concerning cations and anions (Table 7). However, spatial variation in the cations and anions was found to be statistically insignificant (Table 6).

Heavy metal contamination of irrigation water has been recognized as a major environmental threat due to its non-biodegradable nature and extended biological half-life, as well as the potential accumulation in numerous body parts. Residents who consume crops and/or vegetables grown in contaminated areas are exposed to excessive heavy metals due to their deposition in agricultural soils caused by irrigation wastewater (Hussain et al., 2019). In the current study, the heavy metals concentrations in the studied location were within the acceptable FAQ irrigation limits. On the other hand, the seasonal variation was statistically significant (*p* value ˂ 0.05) (Table 7 and Fig. 2).

### Irrigation water quality criteria

#### Sodium adsorption ratio (SAR)

**Fig. 3:**
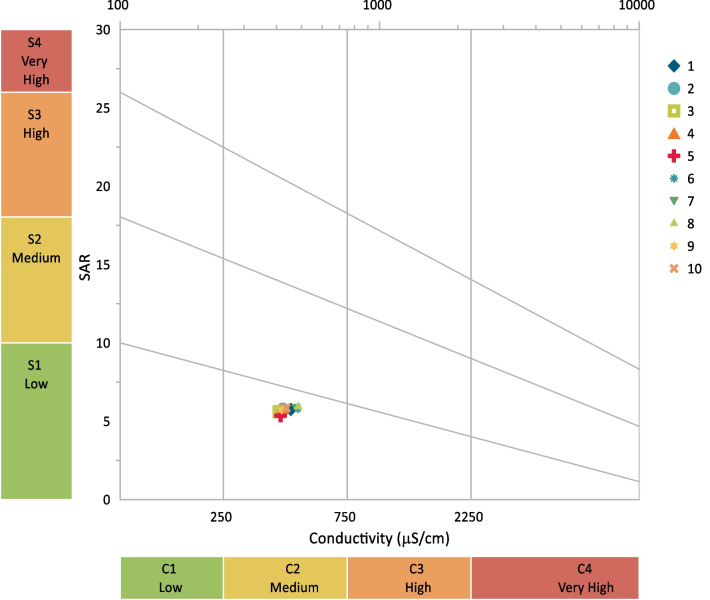
USSL diagram for classifying irrigation waters based on SAR and EC as described by Richards  (1954)

High-sodium irrigation water is particularly problematic because of the negative consequences on the soil. A classification of salt hazards has also been included in the SAR, which may hinder the ability of plants to absorb water (Behboudi et al., 2018). Soil particles absorb Na^+^ and become associated with it. When the soil dries out, it hardens and compacts, making it more water-resistant. For soils with high SARs, certain fertilizers may be necessary. If appropriate concentrations of Ca^++^ and Mg^++^ ions are present in the soil, they will balance the effects of Na^+^ ions and aid in protecting healthy soil qualities (De las Heras & Mañas, 2020). In addition, surface water can be classified as excellent or good (10 to 18), dubious (18 to 26), or undesirable (more than 26) by utilizing SAR, which measures the quality of the water. The samples had a SAR ranging from 5.35 to 5.93 (Table 9). Accordingly, Ismailia Canal sampling sites results were excellent (Table 10). A plot of sample data on the US salinity diagram (Richards, 1954), in which the EC is taken as a salinity hazard and the SAR is taken as an alkalinity hazard (Fig. 3), revealed that all of the samples fell into the medium salinity-low sodium category of water and that they can be used for irrigation on all types of soil with little risk of harmful levels of exchangeable sodium.

**Table 9:** Calculated irrigation water quality criteria including sodium adsorption ratio (SAR), residual sodium carbonate (RSC), sodium percentage (Na%), permeability index (PI%), magnesium hazard percentage (MH%), and Kelly’s index (KI)

Sites	SAR	RSC	Na%	PI%	MH%	KI
1	5.75	− 0.32	39.66	50.91	28.37	0.54
2	5.77	− 0.37	39.68	50.68	28.47	0.54
3	5.60	− 0.35	39.07	50.57	29.81	0.53
4	5.80	− 0.26	39.90	51.20	27.67	0.55
5	5.35	− 0.31	37.99	49.97	32.85	0.51
6	5.80	− 0.33	39.82	50.95	27.92	0.55
7	5.84	− 0.34	40.12	51.17	27.15	0.55
8	5.93	− 0.46	40.07	50.53	27.62	0.55
9	5.72	− 1.25	39.30	48.06	29.26	0.54
10	5.84	− 0.36	39.88	50.83	27.83	0.55

**Table 10:** Categorization of water sites for irrigation utilization based on water quality criteria

Parameters	Site									
	1	2	3	4	5	6	7	8	9	10
SAR	Excellent	Excellent	Excellent	Excellent	Excellent	Excellent	Excellent	Excellent	Excellent	Excellent
RSC	Good	Good	Good	Good	Good	Good	Safe	Good	Good	Good
Na%	Good	Good	Good	Good	Good	Good	Safe	Good	Good	Good
PI %	Moderate	Moderate	Moderate	Moderate	Moderate	Moderate	Moderate	Moderate	Moderate	Moderate
MH%	Suitable	Suitable	Suitable	Suitable	Suitable	Suitable	Suitable	Suitable	Suitable	Suitable
KI	Suitable	Suitable	Suitable	Suitable	Suitable	Suitable	Suitable	Suitable	Suitable	Suitable

#### Residual sodium carbonate (RSC)

RSC is a valuable index for determining the adequacy of irrigation water due to its ability to examine the link between the quantity of carbonate (CO_3_^−−^) and bicarbonate (HCO_3_^−^) and the total Ca^++^ and Mg^++^. Waters containing high concentrations of HCO_3_^−^ tend to precipitate Ca^++^ and Mg^++^ ions when the soil water becomes more concentrated. Soils watered with highly RSC water content may become unproductive due to sodium carbonate accumulation (Khalid, 2019). According to the current results, the RSC values were good for all selected sites (Tables 9 and 10).

#### Sodium percentage (Na%)

**Fig. 4:**
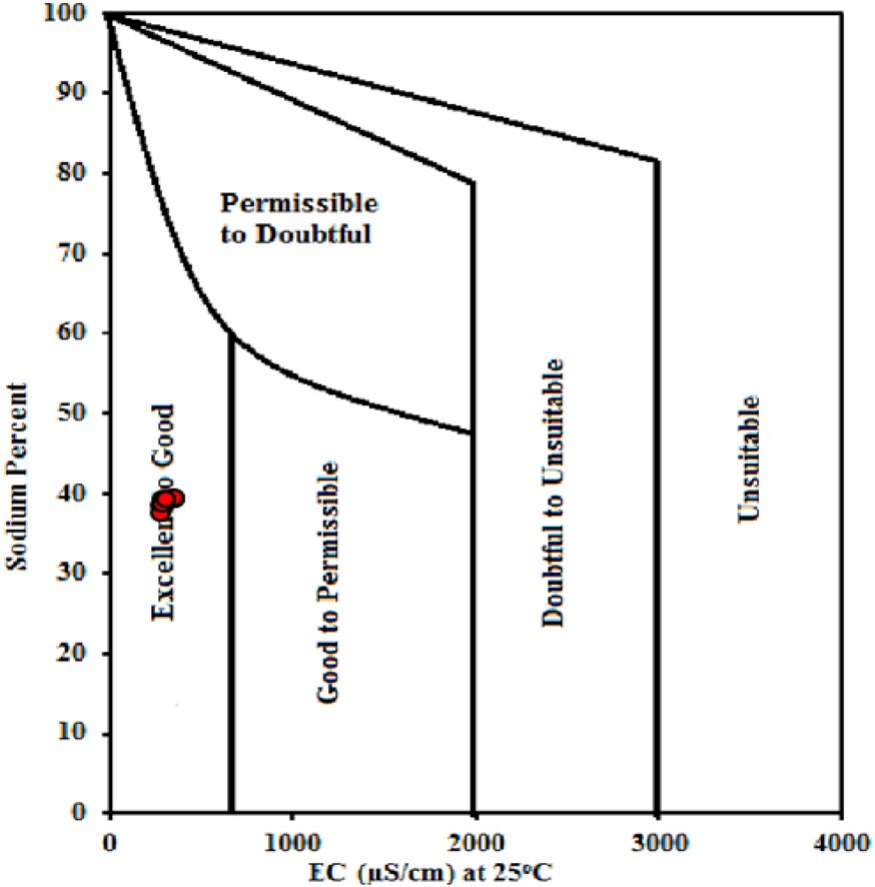
Wilcox diagram illustrating the suitability of surface water for irrigation

The danger of Na^+^ poisoning from surface water can be estimated through calculated the percentage of Na^+^ ions soluble in water content. Na^+^ percentage is a common statistical method used to determine the appropriateness of natural waters for irrigation due to the interaction of Na^+^ ions in the soil and limit its permeability (Shil et al., 2019). More than 60% of water’s sodium content can result in Na^+^ accumulations, leading to soil degradation. Soils with high levels of Na^+^ and CO_3_^−−^ are alkaline, while those with high levels of Na^+^ and Cl^−^ are saline soils (Salifu et al., 2017). Tables 9 and 10 show that all sites along Ismailia Canal exhibited safe Na% levels ranging from 37.99 to 40.12% during the study period. The Wilcox diagram (Wilcox, 1955) relating sodium percent and EC (Fig. 4) revealed that all of the samples were in the “Excellent to Good” range. As a result, the research area’s sites were suitable for irrigation.

#### Permeability index (PI%)

Irrigation efficiency can be measured using the permeability index (PI). The concentrations of Na^+^, Ca^++^, Mg^++^, and HCO_3_^−^ ions are all considered by the PI. The PI value in the research area ranged from 48.06 to 51.20 (Table 9). All collected samples had a moderate PI which increased the irrigation possibility. PI may have increased due to the increased solubility of carbonate and cation exchange in minerals, such as calcite and dolomite (Panneerselvam et al., 2021).

#### Magnesium hazard percentage (MH%)

In most streams, Ca^++^ and Mg^++^ concentrations are in balance. The alkalinity is a phenomenon occurs through soil water intrusion is impeded and crop yields are reduced when Mg^++^ ions and clay particles are present in high concentrations (Omar et al., 2019). Water samples had MH ranging from 27.15 to 32.85%. All tested samples have less than 50% moisture content, making them suitable for irrigation.

#### Kelly’s index (KI)

In the current research, Kelly’s index is used to determine irrigation water quality. This value is derived from the water’s Na^+^, Ca^++^, and Mg^++^ concentrations. Water with a KI value higher than one should be avoided due to its high Na^+^ concentration. KI values in this study varied from 0.51 to 0.55 for a representative water sample (Table 9)**.**

#### Irrigation water quality index (IWQI)

Ismailia Canal water quality index (WQI) was computed using the 10 physicochemical criteria and the weighted arithmetic index approach. Tables 11 and 12 show the parameter scores (*S*_*i*_) as defined by FAO (Lupien, 1994), the ideal value (*V*_*ideal*_), weight (*W*_*i*_), relative weight (*R*_*Wi*_), and IWQI. According to Table 2, the IWQI values varied from 6.84 to 16.7, indicating that the water of the analyzed sample sites is appropriate for irrigation. The discharges of the Misr Petroleum and Abu Zaabal fertilizer companies might affect the WQI values at sites 6 and 9, respectively. The IWQI had a significant positive correlation with the pH (*r* = 0.661) (Table 8). The obtained data also revealed that the drainage water of companies and cities must be thoroughly treated before discharging in the canal to avoid pollution of the canal stream.

**Table 11:** Water quality parameters and their standard values, ideal values, weight, and relative weight

Parameter	Unit	Standard value (*V*_*standard*_)	Ideal value (*V*_*ideal*_)	Weight W_i_ (*1 / V*_*standard*_)	Relative weight (W_i_ / ∑W_i_)
pH		8.4	7	0.119	0.07
EC	μS cm^−1^	3000	0	0.0003	0.0002
TDS	mg L^−1^	2000	0	0.0005	0.0003
Ca^++^	mg L^−1^	400	0	0.0025	0.0015
Mg^++^	mg L^−1^	60	0	0.0166	0.01
Na^+^	mg L^−1^	919	0	0.001	0.0006
Cl^−^	mg L^−1^	1063	0	0.0009	0.0006
NO_3_^−^	mg L^−1^	10	0	0.1	0.06
CO_3_^−−^	mg L^−1^	3	0	0.3333	0.198
SO_4_^−−^	mg L^−1^	960	0	0.0010	0.0006
HCO_3_^−^	mg L^−1^	610	0	0.0016	0.001
Al^+++^	mg L^−1^	5	0	0.2	0.12
Pb^++^	mg L^−1^	5	0	0.2	0.12
Zn^++^	mg L^−1^	2	0	0.5	0.298
Fe^++^	mg L^−1^	5	0	0.2	0.12
				∑ W_i_ = 1.68	∑ Rw_i_ = 1

**Table 12:** WQI and categorization of water sites for irrigation utilization

Site	WQI	Water category
1	9.49	Excellent
2	11.84	Excellent
3	10.18	Excellent
4	9.03	Excellent
5	9.27	Excellent
6	16.7	Excellent
7	10.81	Excellent
8	6.84	Excellent
9	16.37	Excellent
10	9.02	Excellent

As shown in Table 13, the SAR had a high negative correlation with MH% (*r* =  − 0.939), substantial positive correlations with Na% (*r* = 0.951) and KI (*r* = 0.93). In contrast, there was a substantial negative correlation between the Na% and the MH% (*r* =  − 0.988) and a strong positive association between the Na% and the KI (*r* = 0.924). The PI% had a statistically significant negative association with MH% (*r* =  − 0.636). Also, there was a negative correlation between MH% and KI (*r* =  − 0.924).

**Table 13:**
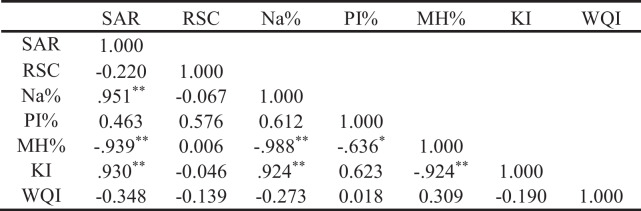
Matrix showing correlation coefficients between the irrigation water quality criteria and the IWQI for the investigated region

*Correlation is significant at the 0.05 level (2-tailed); **Correlation is significant at the 0.01 level (2-tailed)

## Conclusions

According to the classification of irrigation water quality index (IWQI), Ismailia Canal water is acceptable for irrigation according to available data, based on the findings of physicochemical analyses and irrigation criteria. The regional variance between the various research sites was statistically insignificant (*p* value > 0.05). On the other hand, seasonal variation was statistically significant (*p* value 0.05). Therefore, this study recommended conducting studies on the targeted area to shade more light on this important area on a regular and continuous basis in future works to avoid pollution, monitor any change in water quality, determine the effects of pollution on surface water, and determine its suitability for irrigation and human uses. In addition, irrigation water, industrial, municipal, and agricultural drainages must be appropriately treated before blending with canal water.

## Data Availability

The datasets used and/or analyzed during the current study are available from the corresponding author on reasonable request.
